# The role of high-resolution transmission electron microscopy and aberration corrected scanning transmission electron microscopy in unraveling the structure–property relationships of Pt-based fuel cells electrocatalysts

**DOI:** 10.1039/d3qi01998e

**Published:** 2023-12-06

**Authors:** Lazar Bijelić, Francisco Ruiz-Zepeda, Nejc Hodnik

**Affiliations:** a Laboratory for Electrocatalysis, Department of Materials Chemistry, National Insititute of Chemistry Hajdrihova 19 1000 Ljubljana Slovenia nejc.hodnik@ki.si; b University of Nova Gorica Vipavska 13 Nova Gorica SI-5000 Slovenia; c Department of Physics and Chemistry of Materials, Institute for Metals and Technology IMT Lepi pot 11 1000 Ljubljana Slovenia

## Abstract

Platinum-based fuel cell electrocatalysts are structured on a nano level in order to extend their active surface area and maximize the utilization of precious and scarce platinum. Their performance is dictated by the atomic arrangement of their surface layers atoms *via* structure–property relationships. Transmission electron microscopy (TEM) and scanning transmission electron microscopy (STEM) are the preferred methods for characterizing these catalysts, due to their capacity to achieve local atomic-level resolutions. Size, morphology, strain and local composition are just some of the properties of Pt-based nanostructures that can be obtained by (S)TEM. Furthermore, advanced methods of (S)TEM are able to provide insights into the quasi-*in situ*, *in situ* or even operando stability of these nanostructures. In this review, we present state-of-the-art applications of (S)TEM in the investigation and interpretation of structure–activity and structure–stability relationships.

## Abstract 2

Exploring the nanostructures of platinum-based fuel cell electrocatalysts through the lens of advanced transmission electron microscopy techniques unveils the secrets of structure-activity-stability relationships.

## Graphical abstract

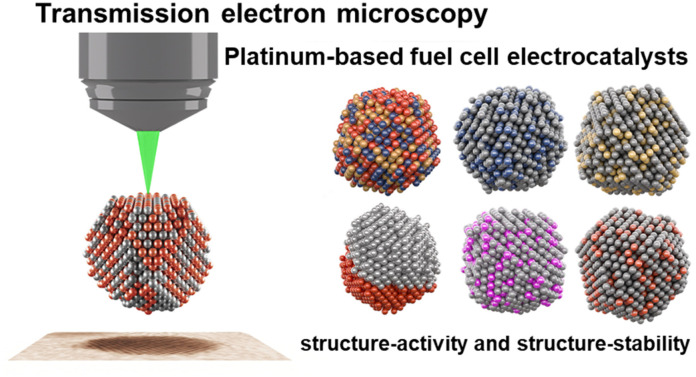

## 1. Introduction

Proton exchange membrane fuel cells (PEMFCs) are considered to have an important role in the transition to clean and renewable energy.^1^ While the hydrogen oxidation reaction (HOR) on platinum nanoparticles is already highly efficient, oxygen reduction reaction (ORR) electrocatalysts are lacking in terms of both activity and stability.^2^ Currently, improving ORR to a desired level requires high loadings of noble metals. The high cost of these noble metals impedes the widespread implementation of PEMFCs.^3^ Interestingly, the annual production of Pt does not limit the commercialization of PEMFCs.^4^ Thus any improvement, in terms of reducing noble metal loading and increasing intrinsic activity and stability, is not only beneficial but necessary for making the renewable energy framework economically competitive with the use of fossil fuels.

Since they display many properties suitable for real PEMFCs, Pt-based materials are a promising class of electrocatalysts for the ORR. Pt exhibits the highest ORR activity among all other pure metals.^2^ On the other hand, the limited availability and the associated high cost of Pt require it to be implemented wisely. These two facts lead to the main challenge in designing Pt-based catalysts, which is to reduce Pt loading while retaining, or in some cases, enhancing the activity and equally importantly stability.^5,6^ To date, various nanoalloys of Pt have been thoroughly investigated. These alloys include metals such as Ni, Co, Fe, Cu, Au, Pd, La, Y and many others.^7–11^ While concerns with demand for metals such as Au, Ni, Co and Y would be somewhat relevant in the large-scale production of nanoalloy catalysts, the abundance of Pt is approximately three orders of magnitude lower than that of these metals (excluding Pd), rendering Pt as the bottleneck in terms of demand and scarcity issues. The cost issue associated with Au and Pd is alleviated through a significant improvement in either the activity or stability of alloyed Pt-based electrocatalysts. The general approach for maximizing Pt nanoalloy activity is based on core–shell nanostructures, whose surface composition is different from that of the particle core. Many of these core–shell nanoalloys have displayed much higher activity than Pt.^7,12^ A subclass of core–shell catalysts involves those with Pt shells that are comprised of a single or a few monolayers, in which case it is often termed Pt-skin.^8^ Another benefit of core–shell structures is that they allow for alloying with some metals that would otherwise easily oxidize and dissolve under the harsh acidic conditions of PEMFCs.^11^ Although this opens up new possibilities for many different catalysts, the stability of core–shell alloy structures remains their biggest weakness. The dealloying of these alloys leads to their inactivation, however, there are cases where controlled leaching is used to obtain so-called Pt-hollow nanoparticles.^13,14^ The principal idea is that only the surface layer directly participates in catalysis, so the main aim of this approach is to reduce the use of Pt while maintaining the activity. In some cases, the activity is even increased, due to strain effects induced by the nature of atoms and structures below the surface. These can also induce surface reconstruction and relaxation.^13^ Besides the composition and structure of these NPs, controlling their size and shape presents another avenue for tuning electrocatalytic properties.^15^ Cubic, tetrahedral and octahedral Pt are just some of the many shapes that these NP alloys can be synthesized.^16^ Additionally, the choice of support is key for ensuring proper dispersion of the catalyst and in many cases, the support also impacts the electrochemical performance. Predominantly, carbon-based supports are used, although many metal-oxide supports are being made and examined as alternatives.^17^

Many catalysts in use today were discovered by a trial and error approach.^18,19^ However, catalysts have been improved to such an extent that it is becoming more difficult to make strides in this field utilizing this type of strategy. The performance of electrocatalysts is fundamentally governed by their structure and composition at the atomic scale.^20^ Therefore, in addition to simply evaluating catalyst performance, it is important that it also gets rationalized by the underlying atomic level structure–activity–stability relationship. One such approach is bottom-up understanding, where we correlate the property of each nanoparticle based on its structure.^21^ When these are established, high-performance catalysts can be predicted and of course, further maximized by fine-tuning their preparation and design.

High resolution transmission electron microscopy (HR-TEM) and aberration corrected scanning transmission electron microscopy (AC-STEM) are powerful and increasingly more relevant characterization techniques in the field of electrocatalysis.^22,23^ Electron microscopy offers invaluable, atomically resolved insights into the morphological, structural, and compositional aspects of catalysts, including changes resulting from activation and degradation.^24^ Ultimately, it can aid in explaining the origin of a catalyst's activity and stability by establishing the structure–property relationships. In order to obtain useful TEM or STEM micrographs, the sample has to be transparent for an electron beam, in a range of approximately below 100 nm in thickness. HR-TEM operates using a parallel electron beam to probe the sample, while STEM uses a focused electron beam that is rastered across the sample.^25^ HR-TEM uses coherent, elastically scattered electrons to form high-resolution (nowadays often with atomic-resolution) images. This is useful for examining particle size, shape, crystal phase and defects. STEM is typically equipped with bright field (BF) and annular dark field (ADF) detectors. BF images are formed by Bragg scattered electrons. ADF images are formed from incoherent, Rutherford scattered electrons which are collected at higher angles, where the scattering intensity is proportional to the atomic number squared (*Z*^2^).^25^ Most (S)TEM microscopes are constructed in such a way that they can be used in both “traditional” TEM and scanning TEM mode. However, there are microscopes solely built for TEM, as well as purely dedicated STEMs. Since STEM has a scanning probe, it's enabled for spatially resolved chemical composition analysis. STEM microscopes are, therefore, commonly equipped with detectors for energy-dispersive X-ray spectroscopy (EDS) and electron energy loss spectroscopy (EELS). Since the implementation of the C_s_-corrector in most STEMs, they typically outshine HR-TEM as the superior choice for most applications, particularly due to their ability to perform chemical analysis and *Z*-contrast imaging.^26,27^ The main downside of STEM compared to HR-TEM is that it's more susceptible to issues arising from scanning distortions and specimen drift, particularly when doing atomic resolution imaging, as well as slower image capturing, which can hinder *in situ* experiments.

By far the most significant advantage of (S)TEM compared to other material characterization techniques, is the fact that it is able to link material nano-structure (atomic structure) to its macroscopic properties, such as electrocatalytic ones. As this review will later cover, the application of identical location STEM (IL-STEM) and *in situ* (S)TEM for this purpose is particularly effective. This is extremely useful for unveiling the relationship between structure and stability, and identifying degradation mechanisms. However, the benefit of being able to analyze the sample in great detail at the scale of a single or a few nanoparticles (NPs) comes with a cost. While (S)TEM provides an abundance of structural and chemical information, properties examined by it are obtained for a small fraction of the sample. Therefore, all microscopic data should be carefully interpreted, as their statistical relevance cannot be guaranteed. Making conclusions based solely on data acquired by STEM is not ideal, and it is good practice to supplement this data with corresponding bulk characterization techniques such as different X-ray analyses: diffraction (XRD), photoelectron spectroscopy (XPS), fluorescence (XRF), absorption spectroscopy (XAS), *etc*.

## 2. Properties of Pt-based nanostructures – conventional (S)TEM analysis

### 2.1. Particle size distribution

**Fig. 1:**
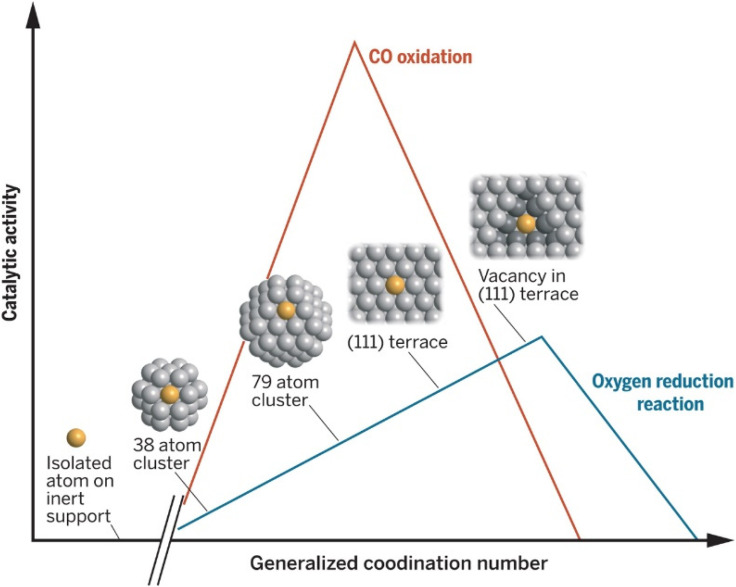
Dependence of Pt catalytic activity for ORR and CO oxidation on the generalized coordination number. The coordination of the active site has a clear impact on the performance of the catalyst. Reproduced with permission ref. 33. Copyright 2015, AAAS.

The electrochemically active surface area (relative to the loading) will depend on nanoparticle size (Fig. 1). Determining the particle size distribution is key when one wants to assess the utilization of metals and how well the catalyst is distributed over the support, especially in the case when investigating highly dispersed systems such as single atom catalysts (SACs).^28–30^ When investigating NPs, another important attribute is the generalized coordination number of surface atoms that was shown to be a descriptor for ORR activity.^31^ The smaller the nanoparticles the more of their surface is occupied by low-coordinated sites, which results in lower ORR activity in the case of Pt. This effect is especially noticeable for Pt-based particles below 5 nm. Interestingly some reports are showing little effect on the ORR activity in that size range.^32^ In general, NP size should affect their electronic structure thereby directly affecting the active sites’ intrinsic activity. For obtaining a particle size distribution, images with a larger number of particles are desirable. Typical procedures involve defining a model for the nanoparticle shape and using software to count the number of particles. It is ideal to find and examine multiple regions of the catalyst with non-overlapping particles. Average particle size can also be determined by XRD and can be used to verify the validity of the (S)TEM-determined size distribution.

A study investigated how different annealing temperatures affect the size distribution of Pt–M alloy NPs.^7^ It's shown that the Pt–M NPs are somewhat larger than commercial Pt/C. The alloys showed a relatively linear dependence of specific activity with respect to average particle size. It was shown that, after degradation tests, the particle size increased, probably as a result of Ostwald ripening or coalescence. Interestingly, the small nanoparticles (<6 nm) grew to a size of ∼6 nm, irrespective of their initial size. The decrease of activity as particles get smaller is a consequence of weaker coordination of surface atoms, which in turn results in too strong binding between Pt and oxygen containing species.^2,34,35^ Yano *et al.* demonstrated how Pt nanoparticle size affects Pt/C electrochemical stability.^36^ The stability of nanoparticles was examined for three different sizes (2, 3 and 4 nm). It was found that, after accelerated degradation tests, the area-normalized activity was retained for all catalysts despite the average particle size increasing. The authors point out the impact of interparticle distance on the resulting activity. For low Pt–Pt particle distances, the O_2_ diffusion fields of multiple particles overlap and the kinetic current density is decreased. It was also shown that Pt/C synthesized *via* the nanocapsule method (n-Pt/C) showed higher stability than commercial Pt/C (c-Pt/C). This was attributed to the high monodispersity of n-Pt/C, which suppressed Ostwald ripening. There are other approaches to improving catalyst stability for ORR, such as dispersing them on N-doped carbon supports.^37^

### 2.2. Morphology and shape

**Fig. 2:**
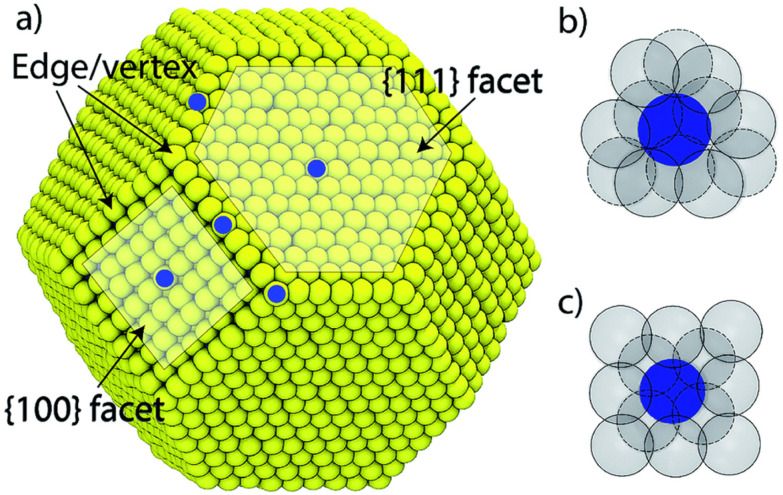
(a) Surface structure of a truncated octahedral nanoparticle. The local coordination structure of a (b) {111} and (c) {100} surface atom. The solid and dashed lines in (b) and (c) denote surface and sub-surface atoms, respectively. Reproduced from ref. 43 with permission from the Royal Society of Chemistry.

Based on the fact that the morphology and shape of nanoparticles can control their surface structure (Fig. 2), such as facets, steps and surface composition, controlling these structural properties can be used to tune electrochemical performance. It is well known that the activity of Pt single crystals is dependent on which facets are exposed to the electrolyte.^38^ In weakly adsorbing electrolytes (*e.g.* HClO_4_), ORR activity on low index facets is shown to be in the order Pt(111) ≈ Pt(110) > Pt(100).^39^ For strongly adsorbing electrolytes, such as H_2_SO_4_, Pt(111) is heavily blocked by sulfates, and the activity trend is Pt(100) > Pt(110) ≈ Pt(111).^40^ The shape of Pt NPs can influence how and which surface facets form.^41^ For example, cubic Pt NPs preferentially terminate with {100} facets, while for tetrahedral particles it's {111} and {110}, and for hexagonal it's {100} and {111}. Wang *el al* show that nanocubic Pt NPs, which have predominately {100} facets, show the highest activity in H_2_SO_4_ compared to NPs of other shapes, such as truncated cubic or polyhedral.^42^ Another interesting finding is that certain high-index facets of Pt exhibit greater activity than low-index ones.^16^

**Fig. 3:**
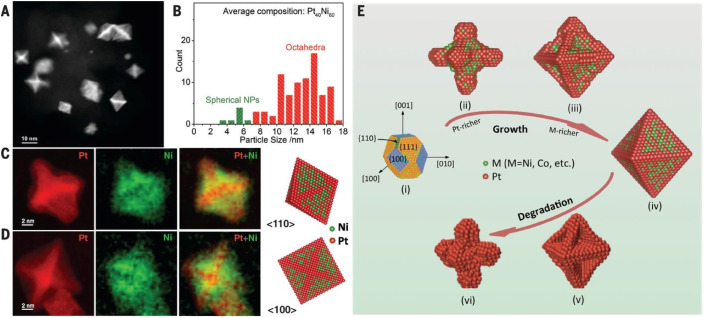
STEM characterization of Pt–Ni octahedral NPs. (a) HAADF-STEM image of the nanoparticles. (b) Particle size distribution, indicating that the average size of the octahedral NPs is ∼13 nm. (c) and (d) HAADF-STEM images (red) roughly representing the Pt distribution and EELS mapping of Ni (green) of PtNi_1.5_ octahedra along [110] and [100], respectively. (e) Illustration of different stages of Pt–Ni nano-octahedra growth followed by the degradation stages. Reproduced with permission ref. 44. Copyright 2014, AAAS.

Concave Pt nanocubes, terminated primarily with {720} facets, were prepared by inhibiting crystal growth in the 〈100〉 direction using a capping agent. The highly active {720}, as well as {830} and {510} facets were confirmed by HRTEM.^16^ One approach to tuning the mass and specific activity of Pt and Pt-based NPs is by synthesizing hollow particles. Using a template removal method, Adzic *et al.* prepared hollow Pt NPs which exhibited higher activity compared to “regular” Pt NPs.^13^ High-angle annular dark-field imaging (HAADF) STEM line scan intensity profiles were used to confirm that the NPs were hollow. Gan *et al.* performed an analysis of octahedral Pt–Ni NPs and established that they are found in both branched and near-spherical shapes.^44^ The formation of octahedral particles was attributed to their low surface energy, while the branched NPs formation was explained by kinetically favorable anisotropic growth (Fig. 3). After a certain critical particle size was reached, anisotropic growth dominated surface energy minimization, resulting in hexapod-like particles. Pt_3_Ni alloys doped with Mo, also synthesized in an octahedral shape, display a tremendous activity increase of approximately 80 times compared to commercial Pt/C catalyst.^22^

**Fig. 4:**
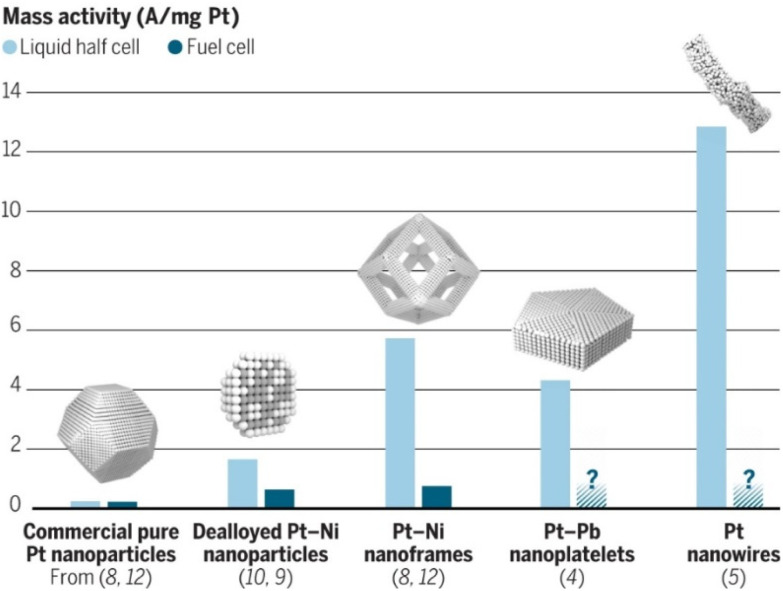
A comparison of ORR mass activity of different Pt and Pt-based catalyst nanostructures. The activities of more intricate nanostructures exceptionally decrease when comparing an experimental half cell to MEA, indicating that additional challenges arise in real fuel cell implementation. Reproduced with permission ref. 48. Copyright 2016, AAAS.

The work of the Stamenkovic group on polyhedral Pt_3_Ni nanoframes is another example of how tuning catalyst shape can enhance its activity and durability.^45^ The increased activity is ascribed to the high surface area of the hollow nanoframe structure, as well as ligand and strain effects imposed on the Pt shell. The specific activity of the Pt_3_Ni nanoframes was over 16 times higher than the commercial benchmark Pt/C. The remarkably high stability of the nanoframes was credited to the modified Pt shell electronic structure, which weakens oxygen binding strength and reduces the rate of Pt dissolution. Apart from the mentioned NP shapes, many other nanostructures, such as jagged nanowires^46^ and nanoplates,^47^ demonstrate enhanced ORR activity and stability. Still, there are issues in transferring these high ORR activities to application-relevant environments (*i.e.*, MEA), as can be seen from Fig. 4.

### 2.3. Strain

It is now well established that the electronic structure of a material strongly influences its electrocatalytic activity.^49–51^ Since the development of the d-band centre theory, the performance of alloys is often explained in terms of two main phenomena arising when metals are alloyed, the ligand and strain effect. The ligand effect refers to the inherent change of electronic structure when combining the base metals to form an alloy, as a result of different electronic configurations of the metals. The strain effect refers to the influence of change in interatomic distances on the electronic structure. Tuning catalyst activity by exploiting the ligand and/or strain effect is a popular approach to designing highly active alloy electrocatalysts.^52,53^ It is generally considered that compressively straining Pt leads to an increased activity towards the ORR as a result of lowering the d-band and weakening the adsorption of oxygen-containing species.^54^

While strain measurements can be performed by XRD,^55^ STEM and HR-TEM offer local visualization of the strain field which is beneficial to assess which part of the NPs has an effect on its catalytic properties.^56^ There are many strain determination methods that can be applied by both HR-TEM and STEM. The simplest approach to determine strain is by directly measuring distances between the atomic columns of a nanoparticle in multiple regions and comparing the *d* values. This approach is straightforward, however it is somewhat impractical and imprecise, and also cannot be used for getting a complete strain map of the particle(s). In general, strain mapping methods can be classified into two groups: based on high resolution images and based on diffraction patterns. Strain maps can be extracted from high resolution images in four main ways: peak-finding (PF) method, peak-pair (PP) method, dark field electron holography (DFEH), and by geometric phase analysis (GPA). The first three methods analyze the images in real space, while GPA utilizes reciprocal space. With the PF method, atom positions are fitted with 2D Gaussian functions. An unstrained reference lattice (internal, from the core of the particle) is then extrapolated and compared to the real, strained lattice. The strain is calculated based on the displacement of the real lattice relative to the extrapolated, ideal lattice. Using this method, Gan *et al.* showed increased activity in partially dealloyed PtFe nanoparticles as a result of a compressively strained Pt-rich surface.^57^ By modelling PtFe NPs with different distributions of Pt and Fe, and comparing simulated strain values to measured ones, they concluded that the dealloyed particles have a Fe-rich core and a Pt-rich shell.

Similarly, the PP method uses pairs of peaks in atomic columns to compute interatomic distances and calculate strain by comparing them to an unstrained region.^58^ The main advantage of PP and PF over GPA is that they are less computationally demanding and may be better suited for smaller regions. DFEH strain analysis is done by passing one portion of the electron beam through a reference, unstrained area and another through the region of interest, after which the beams interfere with a biprism to form a hologram.^59^ The resulting beam carries phase information about the sample, and by using Bragg filters, strain can be extracted. The biggest advantage of DFEH over other techniques is it enables capturing strain maps with a wide field of view (FOV) while preserving high strain sensitivity. The disadvantage is that a biprism and a Lorentz-type lens are necessary. It should be noted that, for very small nanoparticles, the ability to pass the beam through a strained and non-strained region which are isolated from one another may not be possible.^59^

**Fig. 5:**
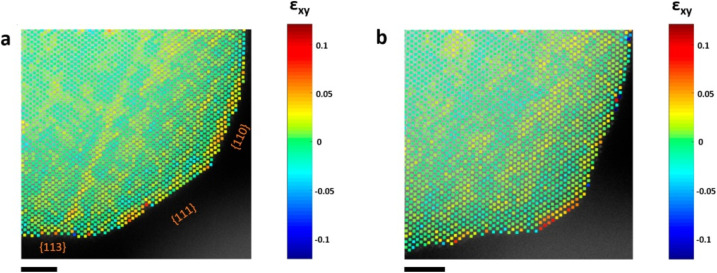
Strain analysis of a PtCu_3_ nanoparticle. Shear strain (*ε*_*xy*_) map (a) before and (b) after electrochemical activation.^60^ Open access, 2019.

Geometric phase analysis (GPA) employs algorithms that use fast Fourier transform (FFT) to analyze images. Local strain fields are calculated from two non-collinear diffraction spots selected in the FFT of the image.^61^ The main advantage of GPA is that the measured strain is less affected by optical aberrations in (S)TEM, because the information is contained in a local region of reciprocal space. The drawback of GPA is its requirement that the images are very high resolution (*i.e.* particles oriented on zone axis). An example of a strain map obtained with GPA is shown in Fig. 5. Gamler *et al.* used GPA to map strain in Pd@Pt, Rh@Pt and Rh@Pd core–shell nanoparticles.^62^ They show that the shell lattice of Pt in Pd@Pt is barely deformed relative to the Pd core, meaning that the Pt shell is compressively strained to match the lattice of the core. A similar effect was observed for Rh@Pt and Rh@Pd. For the same particles prepared with thicker shells, strain maps showed expansive relaxation (relative to the core). Shell relaxation is a result of the weakening of strain effects with distance from the core–shell interface. Shell expansion for the thicker shell particles was expected, as all three of the investigated core–shell NPs have shells containing metals with larger lattice parameters than the metals present in the particle cores.

Like in the case of high-resolution image-based strain mapping, obtaining strain maps from diffraction patterns can be done in numerous ways. Key diffraction-based methods include selected area electron diffraction (SAED), convergent beam (CBED) and nanobeam electron diffraction (NBED). In SAED and NBED, lattice parameters and their deviations are measured based on diffraction spot positions (relative to the central spot).^63^ The main difference between the two is that SAED uses an aperture to select a region of the sample, while NBED utilizes a nano-sized (nearly) parallel electron beam. SAED has ∼2% error in determining lattice parameters,^25^ so in some cases, it is more suitable for determining the presence of different phases where there are larger variations of lattice parameters, than for accurate determination of strain. Strain mapping using NBED is more popular due to its higher precision and sensitivity compared to SAED.^64^ While the resolution in SAED is limited by the aperture size (∼μm), in NBED the beam size (∼nm) defines the resolution limit. In this case, a larger field of view can be analyzed compared to high-resolution image-based methods. However, dynamical diffraction may cause difficulties in accurate identification of the diffraction disks or spots. CBED, on the other hand, can be used to determine strain to a much higher precision, as lattice parameters can be determined with an accuracy of ∼0.01%, by examining the structure of the disks in the diffraction pattern.^63^ The disks form as a result of combined Bragg scattered electrons and multiple scattering electrons. The diffraction disk contains High order Laue zone (HOLZ) lines, whose position and width are sensitive to lattice parameter values.^65^ The main disadvantage of CBED is that analysis of the diffraction disks can be complex and in some cases for thin samples and nanoparticles the HOLZ lines are undetectable.^66^

SAED strain measurements were used to explain improved activity for ORR, demonstrated on biaxially strained Pt/PtPb nanoplates.^47^ The phases of PtPb and Pt were examined by comparison of experimental with simulated images, confirming a hexagonal PtPb and cubic Pt core–shell structure. They explain that the formation of a biaxial strain, which is compressive along [011] and tensile in the [100] direction, results in a large increase in ORR activity. Even though there is generally a consensus that compressively strained Pt should have enhanced kinetics, this finding shows that the approach of compressively straining Pt may not always result in favourable performance. Interestingly, they also show that nanoparticles of the same composition show a much smaller activity increase (in reference to benchmark Pt/C) than the Pt/PtPb nanoplates.

### 2.4. Composition

The elemental composition of NPs can be determined by energy dispersive X-ray analysis (EDS) and electron energy loss spectroscopy (EELS). The major advantage these two techniques have over other methods for elemental composition measurements is the capability for local, spatially resolved analysis. Both techniques are usually performed in STEM mode, as they require a converged probe in order to map a localized feature. It should be noted that, EELS can be used for mapping using energy filtered TEM (EFTEM). Despite this, EELS mapping in STEM mode is a more common approach. The composition can be determined either as line profiles (1D distribution) or by mapping (2D distribution). In general, EDS is better suited for analyzing heavier elements, while EELS is better for lighter elements.^67^ In terms of spectrum acquisition and mapping, EDS generally requires less expertise from the user than EELS and the interpretation of EDS results is also more straightforward. EDS has an energy (spectrum) resolution of ∼120 eV.^20^ This resolution is satisfactory for yielding qualitative and quantitative information about the composition, however, it is not possible to examine more detailed information such as the chemical environment (chemical shift), valency, spin states and other similar properties. On the other hand, EELS, when using a field emission gun, can have an energy resolution of ∼0.7 eV. If the electron gun is equipped with a monochromator, it can reach 0.2 eV. Newer designs have made it possible to achieve a resolution of 4.2 meV.^68^ Another factor that needs to be considered is sample thickness. EDS can be performed independent of specimen thickness, while EELS requires thin samples in order to avoid multiple scattering events, which results in poor S/N and a large background. The thickness of the sample should ideally be equal or lower than the inelastic mean free path of the given material, which in the case of most metals is approximately 50–100 nm.^69^ EDS is extensively used for elemental composition mapping of alloy NPs in the field of electrocatalysis. For example, it can provide an easy assessment of whether the examined alloy NPs have metals homogenously distributed^70,71^ or if a core–shell structure is formed.^72^ Sasaki *et al.* deposited a Pt monolayer on PdAu NP cores *via* galvanic displacement.^73^ EDS was used to measure the distribution of the three metals and confirmed a higher Pt concentration at the particle edges. The authors point out the importance of the inclusion of a small amount of Au and its role in increasing electrochemical stability. Hunt *et al.* established a self-assembly method for controlled synthesis of transition metal carbide (TMC) NPs coated with a Pt shell.^74^ They investigated the stages of Pt/TMC nanoparticle formation by STEM. EDS line scans were used to determine the distribution of Pt on WC, confirming that Pt is localized at the shell of the particles. Xie *et al.* synthesized Pt-coated Pd nanocubes with tuneable Pt shell thickness.^75^ Using different Pt precursor concentrations and temperature treatments, shells comprised of 1–6 atomic layers of Pt were confirmed by STEM-HAADF images and EDS line scan profiles of Pt and Pd. They demonstrated that, out of all the coated NPs, those with just a monolayer of Pt show the highest mass activity.

EELS has a wide range of applications for the characterization of NP samples. Low-loss EELS spectra, corresponding to phonon and plasmon excitations, provide information about sample thickness, band gaps and electron density. While this information can be useful, the core-loss region is of greater interest for Pt-based ORR catalysts. The elemental composition of the metals in nanoalloys, as well as their chemical environment can be probed. In addition to investigating the catalyst, EELS can give valuable chemical information regarding the support. For carbon-based supports, structural information can be obtained from the carbon K-edge,^76^ while for metal-oxide supports, valence states can be examined from the intensity ratio of L_3_ and L_2_ lines and their respective edge thresholds.^77,78^ A potential drawback could be high beam exposure and thus altered and damaged specimen structure.

A study investigated the distribution of Pt and Co in Pt_3_Co/C core–shell NPs.^14^ They demonstrate that the NPs have a Pt-rich shell and an alloyed Pt_3_Co core. Additionally, the change in particle composition with aging was studied. The particles were found to have three distinct possible structures after aging: Pt–Co core–shell NPs with a thicker Pt-rich, Pt–Co “hollow” NPs with reduced Co content and pure Pt “hollow” NPs where Co from the core is completely dissolved.^14^ Another study used EELS to investigate the compositional variation of Pt_*x*_Ni_*y*_ octahedral NPs.^79^ EELS line scans provided evidence of Ni leaching preferentially at the {111} facets, resulting in concave octahedral particles. The magnitude of change in the shape was shown to be dependent on Pt : Ni ratio, and was most evident for PtNi_1.5_. The activity, and how it changed during the lifecycle of the particles, was shown to be governed by Pt shell thickness, subsurface Ni concentration and fraction of remaining {111} facets. Another interesting and recent application of EELS was to analyze the ionomer.^80^ Previously this has been done by labeling/staining the ionomer with Cs and Ba ions and using EDS to map the ionomer coverage on NPs.^81^ The main disadvantage of this approach is that it does not guarantee homogenous staining. EELS bypasses the need for labeling of the ionomer because carbon in the support and ionomer have a different C K-edge structure. Vulcan XC72 gave rise to a π* edge, originating from sp^2^ bonding in amorphous carbon, while this feature is absent for Nafion. In addition, a shift in σ* edge position was observed. Based on edge positions, it was concluded that Nafion gives rise to σ* (C–C) and (C–F) peaks, at higher energy losses compared to Vulcan XC72 σ* (C–C) edge.^80^

## 3. Deep insights into Pt-based nanostructures – advanced (S)TEM techniques and methods

### 3.1. IL-STEM

Identical location STEM (IL-STEM) is a method where samples are analyzed before and after an electrochemical or, more generally, any treatment of the specimen. This is done using a TEM finder grid as the sample substrate, where the grid is labeled in some way (*e.g.* alphabetically). We note that also classical TEM grids can be used. The sample is first imaged and characterized prior to any electrochemical experiments. The grid is then transferred to either a rotating disk electrode (RDE) setup or a modified floating electrode (MFE) setup. The sample is subjected to any desired treatment (*e.g.* accelerated degradation test) after which it is transferred to the microscope and analyzed again. This procedure can be repeated multiple times, for example, to identify different stages of catalyst degradation.^82,83^ IL-STEM measurements are of paramount importance in elucidating how catalyst structure, chemical composition and morphology change with degradation, and can help pinpoint the “stability bottleneck” of a particular catalyst. At atomic-resolution, IL-STEM can help in the bottom-up understanding of degradation mechanisms at a more fundamental level and thus establish structure–stability relationships.^21^

**Fig. 6:**
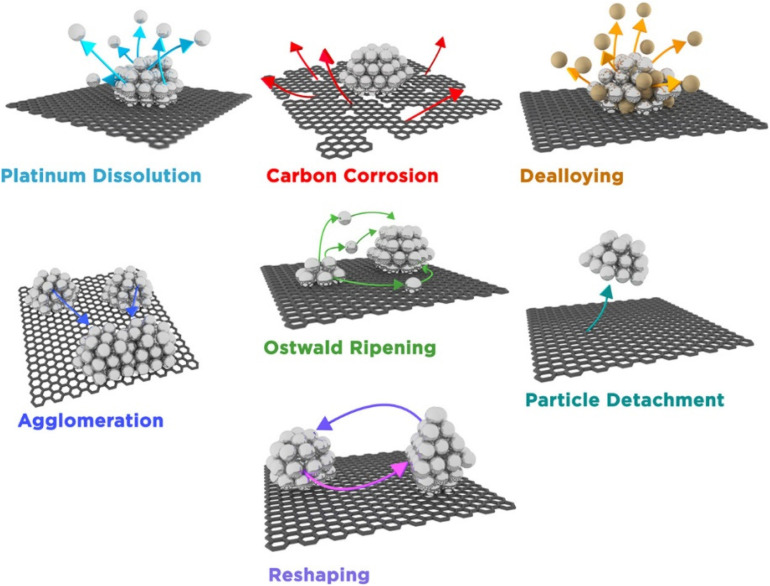
An illustration of different degradation mechanisms of Pt nanoparticles supported by carbon.^83^ Open access, 2016.

**Fig. 7:**
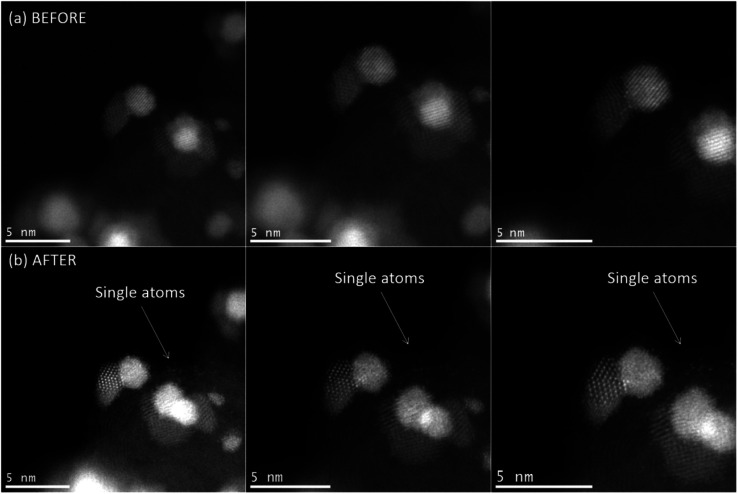
IL-STEM images of Pt–SnO_2_/C nanoparticles (a) before and (b) after 1000 electrochemical cycles from 0.05–9.8 V (*vs.* RHE) at different magnifications. Single atoms appear after degradation and are shown to not move from beam exposure. Reprinted with permission from *J. Phys. Chem. C*, 2018, **122**(18), 10050–10058.^96^ Copyright 2018, American Chemical Society.

For Pt-based NP catalysts, it is generally considered that there are multiple different degradation mechanisms (Fig. 6) that lead to loss of activity.^83^ How prominent a specific degradation mechanism is will depend on the catalyst material and working conditions such as pH, temperature, and electrochemical parameters (*e.g.* potential window for cycling). One study demonstrated that the extent of Pt degradation is more dependent on the number of potential cycles than on the scan rate.^84^ For Pt-based materials, the most common degradation mechanisms are particle detachment,^85,86^ dissolution,^84,87,88^ Ostwald ripening,^89^ agglomeration,^90,91^ and support corrosion.^90,92^ All mechanisms have in common either a loss of active material or an increase in particle size. For a more comprehensive review on degradation mechanisms and how they are examined with IL-(S)TEM, the reader is referred to ref. 93. An atomic resolution IL-STEM study of Pt–Ni NPs revealed that they preferentially dissolve on steps and corners and that Pt redeposition occurs predominantly on {111} facets.^94^ Particle size distributions often reveal the dominant degradation mechanism. An average particle size increase with a tail towards smaller particle sizes is indicative of Ostwald ripening. On the other hand, the appearance of a tail towards larger particle sizes is typically a result of agglomeration. If the size distribution doesn't change much with degradation, it is considered that detachment is the primary mechanism. An example of how the particle size of Pt/C NPs is affected by potential cycling is given in ref. 95. Another possible consequence of degradation is the appearance of single atoms (Fig. 7).

**Fig. 8:**
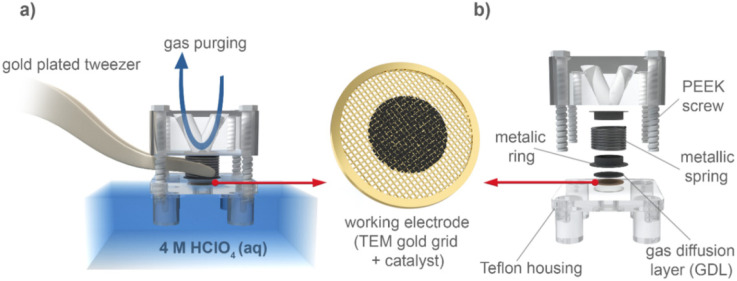
An illustration of the modified floating electrode (MFE) setup.^97^ Open access, 2020.

A modified floating electrode (MFE) setup has also been used for IL-STEM experiments^21,97,98^ (Fig. 8). In the MFE setup for the ORR, the electrode is supplied with O_2_. As opposed to RDE where the reaction takes place at the catalyst/electrolyte interface, for MFE the reaction takes place at the gas/catalyst/electrolyte interface. This inherently results in enhanced mass transport and higher, industry-relevant currents. The MFE setup takes advantage of the relatively straightforward use of TEM grids as a backing electrode for catalyst deposition, allowing for direct IL-STEM. This setup was further upgraded to the so-called *Nano Lab* concept.^99^ The idea of Nano Lab is to use anodic oxidation of the TEM grid as a synthesis method, followed by IL-STEM characterization of the grid. After this, electrochemical characterization can be performed and IL-STEM can be used again, to investigate the impact on the catalyst and support.^21^ This method is also complementary to other characterization techniques, such as XPS, Raman spectroscopy or IL-SEM.

**Fig. 9:**
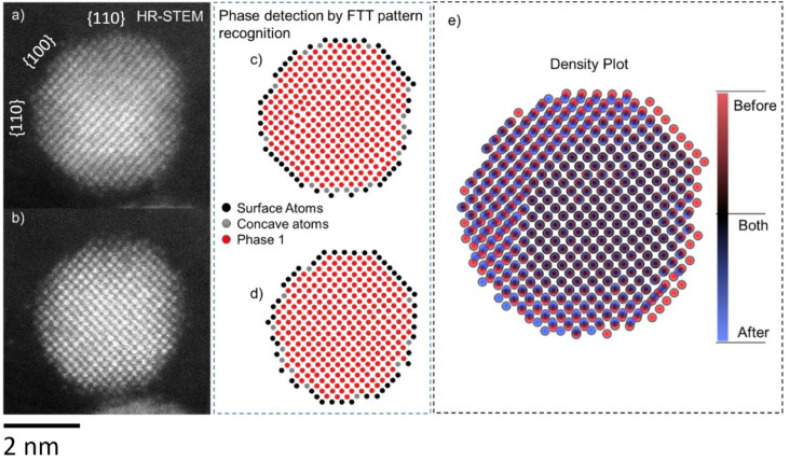
IL-STEM images of a Pt–Co nanoparticle (a) before and (b) after electrochemical activation. Atomic positions obtained *via* phase detection using FFT patterns, (c) before and (d) after activation. (e) Density plot of atomic positions before and after, overlayed.^98^ Open access, 2021.

An important part of identical location measurements is the image processing and data analysis that comes after. As a means to aid in improving image quality, denoising and extracting quantitative data, automated image analysis for TEM is highly useful. Examples of what can be obtained using image analysis algorithms, such as determining atomic column positions (Fig. 9), strain analysis and 3D reconstruction, are given in ref. 100.

### 3.2. Electron tomography

Investigating sample morphology is often done by scanning electron microscopy (SEM), since secondary electrons contain surface information from the interaction of the sample with the beam, dependant on the accelerating voltage. Although the possibility to couple it with an aberration corrected STEM as an optional feature exists,^101^ when a full description of the inner structure and further resolution is necessary, including surface morphology, as is the case for samples of small NPs, the use of (S)TEM is required.^102^ TEM-based techniques give a 2D projection of a sample with 3D structure, so the morphology cannot be examined directly and different projections are necessary to construct a model.^102,103^ However, a full 3D image can be reconstructed from a set of 2D images obtained at different tilt angles (orientations) of the sample.^104^ This method is known as electron tomography (ET).

Shape and morphology studies in IL-TEM^105,106^ can also benefit from tomography. In order to visualize the 3D arrangement of nanoparticles and the support, before and after degradation, IL-tomography was performed on a Pt/C catalyst before and after degradation.^90,107^ Particle growth and change of the 3D shape of the carbon support, indicating carbon corrosion, are both clearly seen from the comparison of IL-tomography images before and after degradation. It was shown that, after degradation, a higher fraction of Pt particles are retained in regions where multiple carbon particles are in contact, compared to more exposed carbon regions.^90^ As a prospect, it could, for instance, reveal the surface coordination of all atoms and their stability if performed with atomic resolution,^108^ and even information related to the local orientation and the bond length,^109^ as well as 3D strain distribution.^110^ Alternatively, in this regard, 3D information from a single STEM ADF projection is possible if the material is composed of a single element,^111^ but when two or more elements are in play, new approaches are needed.^112^ 3D chemical distribution maps have also been part of the developing techniques in an effort to try and investigate the complete picture of the material despite certain limitations, such as the signal-to-noise ratio.^113^ Other issues include the long acquisition times, which represent a drawback for the applicability of the technique in beam sensitive materials or during the impletmentation of *in situ* experiments.^114^ Up until now, successful reconstructions of Pt/C fuel cell catalyst nanostructures have been achieved, where ADF tomography and inpainting algorithms were employed to improve the visualization and hence mitigate common artifacts.^115^ Automatization has also been part of the current tomography developments, when investigating Pt catalysts before and after accelerated stress testing,^116^ allowing for a better statistical analysis as the size of sampling increases, in analysing and determining degradation in particles that are either inside pores or outside the carbon support. Additional examples involve the use of cryo-electron microscopy to reduce beam damage and determine the morphology of layer aggregates, including the ionomer layer and the location of Pt on carbon supports.^117^

### 3.3. 4D STEM

4D STEM is a sub-technique of STEM that consists of capturing a 2D diffraction pattern with a fast camera at each probe position of a STEM image.^118^ The name comes from the fact that each pixel in a 2D image contains a complete 2D diffraction pattern. 4D STEM can be used for very accurate strain mapping by NBED.^64,119^ An example of strain mapping using 4DSTEM is shown for core–shell Rh@Pt nanocubes.^120^ In this work, a comparison of strain mapping using GPA, atomic column tracking and 4DSTEM nano-diffraction are given. The authors establish some key advantages of 4DSTEM, such as a lower variance of the measured strain compared to the other two methods. The method is also more robust against drift and scan-distortion effects compared to the other two methods. With the implementation of scanning NBED, crystal phase maps and orientation maps can be obtained.^121^ Furthermore, the use of precession electron diffraction has also been shown to enhance the clarity of the diffraction patterns, helping in a better and easier identification of the crystal structure.^122^ Centre of mass (COM) and differential phase contrast (DPC) analysis of diffraction patterns can highlight sample features that are of interest. COM analysis, for example, can be used to obtain electric field and charge distribution maps.^123^ This is particularly useful for investigating catalyst–support interactions, in cases where charge transfer between the two is significant. The structure and chemical composition of the catalyst/support system are frequently shown to govern the electronic structure and the resulting activity and stability of the material.^124–126^ Zachman *et al.* established a protocol in which 4DSTEM is used to map the charge distribution on an Au/SrTiO_3_ catalyst/support system.^127^ The direction of charge transfer was shown to be independent of particle size. Although a net negative charge was measured on Au particles, individual atoms of Au close to O atoms of the support were positively charged. It was also demonstrated that the charge transfer direction can be inverted using post-synthesis treatments. Tuning catalyst activity, selectivity and stability by controlling catalyst–support charge transfer is, as of now, a far more widespread method in the field of heterogeneous catalysis than in electrocatalysis, leaving a lot of room for studying ORR electrocatalysts with this kind of approach. Another interesting phase imaging technique that derives from 4DSTEM is electron ptychography, which enables enhanced imaging reconstruction^128^ and a more accurate structural representation using low beam doses. This facilitates theinvestigation of beam sensitive materials^129^ and theimaging of light and heavy elements at the same time.^130^ In addition, electron ptychography has been used in combination with depth sectioning for 3D reconstructions.^131^ Alongside, a recently developed method named bright-field balanced divergency (BBD), has shown significant advantages as a non-iterative, dose-efficient and noise-robust reliable way for phase retrieval, being complementary to current phase imaging approaches.^132^

### 3.4*. In situ* (S)TEM

The previously mentioned applications of electron microscopy only deal with analyzing the catalyst at given points during its lifecycle. *In situ* (S)TEM provides information about how the catalyst is influenced by given conditions and the structural changes it undergoes during reactions.^133^ This is extremely useful as it eliminates the need for any speculation about the intermediate states of the catalyst material. *In situ* (S)TEM, compared to other analytical *in situ* techniques, is distinguished by its capability to monitor the morphological evolution of the electrocatalyst during either crystal growth or electrochemical reactions.^134^ The most common forms of *in situ* TEM used for electrocatalysts are liquid and gas atmosphere environmental TEM (ETEM). Liquid *in situ* TEM can be realized either by differential pumping^135^ to increase the pressure sufficiently in order to keep the liquid from evaporating or by using a closed-cell setup.^136^ In this regard, spatial and temporal resolution play an important role when imaging dynamic processes, and additional effects arising from sample thickness and stability need to be considered.^137^ One of the first *in situ* liquid cell TEM studies analyzed the nucleation and growth of Cu *via* electrodeposition.^138^ The cell used was constructed as two Si wafers, separated by a SiO_2_ ring and etched to create a window on which Au working electrode was deposited.

An *in situ* study of octahedral Pt–Ni alloy NPs, using an electrochemical liquid cell TEM chip showed that, under a high potential, the particles rapidly agglomerate preferentially along {111} facets.^139^ Large (20–50 nm) Ni-rich alloy particles displayed nearly complete dissolution, as opposed to octahedral (∼8 nm) particles partially protected by surface Pt. It was also revealed that startup/shutdown potential changes accelerate coarsening of the catalyst. Carbon corrosion was shown to be far more prominent during a potential hold at high potential, compared to potential cycling. Gong *et al.* conducted an analysis of the influence of heating atomically disordered PtCu nanoframes on their restructuring and formation of ordered intermetallic PtCu.^140^ In addition to this, by coupling a tomography approach with EDS, the distribution of Pt and Cu was mapped in 3D during the ordering of the nanoframes. It was concluded that while this reduces leeching of Cu, it leads to aggregation of the nanoframes.

Although without electrochemical control, Liao *et al.* investigated Pt nanocube growth and the kinetics of facet growth.^141^ The nucleation and growth of particles was stimulated by beam irradiation. At early stages of growth, all three low index facets ({100}, {110} and {111}) grew at similar rates. When particle size reached 2.5 nm, the {100} facet ceased to grow. This effect is contrary to thermodynamic considerations, such as what would be expected based on Wulff construction.^142^ This inhibition of {100} growth was explained by taking into account kinetic effects, involving different ligand (surfactant) hopping energy barriers on different facets. The anisotropic nature of structural changes of Pt is also revealed to be present in cathodic corrosion, demonstrated on Pt single crystals in a H_2_ gas cell.^143^ The formation of Pt_3_Fe nanorods from nanoparticles has been studied by liquid cell *in situ* TEM.^144^ The nanoparticles first formed *via* beam induced reduction of Pt and Fe precursors. The interactions of these nanoparticles then lead to formation of chains. It was demonstrated that inclusion of an additional surfactant results in straightening of NP chains into single-crystal nanorods. Increasing the resolution of *in situ* (S)TEM was demonstrated through an approach utilizing a graphene liquid cell.^145,146^ An MoS_2_ monolayer separated by hexagonal BN from graphene cell windows was developed and used for monitoring the adsorption and diffusion dynamics of Pt atoms on MoS_2_.^145^

ETEM, while not directly of interest from the perspective of electrocatalysis, is highly relevant for studying the structural dynamics during the synthesis of electrocatalysts. Synthesis of PtCo/C NPs in a H_2_ atmosphere was monitored *in situ.*^147^ The study revealed that during annealing of Pt and Co precursors, the formation of small (<3 nm) and ordered tetragonal PtCo particles begins at 450 °C. Despite this, the majority of particles at this stage were disordered FCC alloys. At temperatures above 700 °C, completely ordered FCC PtCo and Pt_3_Co NPs are formed, with particle sizes >4 nm. After reduction at 700 °C, the number of smaller NPs drastically decreased, signifying that Ostwald ripening and agglomeration had occurred. The average size of the synthesized particles had increased roughly three times compared to precursor particle sizes.

It is important to note that *in situ* TEM conditions that can currently be realized are far from real working conditions.^74,148,149^ The main reason for this is the fact that high gas pressures in gaseous TEM cells, and thick liquid layers in liquid TEM cells are undesirable, as the electron beam would suffer too much scattering from the medium.^150^ From the electrochemical point of view, liquid cell electrochemical TEM is still not capable of replicating bulk electrochemistry quantitatively, as a consequence of the size constraints of liquid cells,^149^ which leads to different cell geometry and non-uniform electric fields during operation.^151^ Beam-induced radiolysis was also shown to change the local redox chemistry of an aqueous system.^152^ The beam sensitivity of both the sample and surrounding medium are also a concern and this can be the main limitation for experiments that require long exposure times.^148^ An overview of beam induced effects on electrochemical systems is given in ref. 153.

It must be emphasized that adding electrodes to the liquid TEM cell and thus controlling the potential of the specimen is still considered a big challenge. However, with the great progress in cell designs and sufficient lowering of the beam dosage, this should in principle be possible now. In addition, carrying out an operando and a multimodal analytical approach, there have been relevant experiments revealing new insights regarding degradation in nanocrystals.^154^*In situ* TEM has been applied with great success in neighbouring research areas such as synthesis and crystal growth,^155^ heterogenous catalysis,^156^ corrosion^157^ and batteries.^158^ On the other hand, the most recent, and groundbreaking improvements of *in situ* set-ups, are yet to be fully utilized for characterization of Pt-based ORR electrocatalysts at reaction conditions. Surprisingly, a relatively high number of studies concerning Cu-based catalysts for CO_2_ reduction using *in situ* TEM have been reported, such as.^159–161^

Lastly, with the recent capability to implement artificial intelligence tools in the analysis of microscopy data,^162,163^ namely, in structural imaging, chemical analysis, and momentum-resolved (4D) STEM, new opportunities arise for improvement and automation, from enhancing imaging in structural dynamics videos,^164^ to planning experiments, including the manipulation of matter at the atomic scale.^165^ Nevertheless, one of the goals currently being solved is to be able to handle large amounts of data, required to train models, and the creation of an effective interface to interpret the information in a friendly manner.^166^

## 4. Summary

PEMFCs play a vital role in the renewable energy framework. Despite many achievements in the field of ORR electrocatalyst research, the main goal of a highly active, stable, and affordable catalyst is yet to be realized. While there are several reports on Pt-based nanocatalysts which fulfill the requirements in terms of activity, the stability of these materials often remains mediocre, while the cost is still relatively high. The performance of electrocatalysts is governed by their atomic structure. At present, approaches that consider synthesis optimization in order to control nanocatalyst structure and thus tune its activity seem to be the best course of action. Combined with electrochemical benchmarking experiments, the use of (S)TEM to probe the atomic structure is vital in order to achieve a more thorough understanding of the underlying structure–activity–stability relationship. Degradation phenomena, particle size effects, shape and facet-dependent behavior, catalyst–support interactions, strain induced effects, elemental distribution and more fine chemical information are all properties that can readily be investigated by multimodal analytical transmission electron microscopy. Insights into electrochemical degradation and the catalyst–support interactions seem to be of the highest importance in unraveling the influence of structure and composition on activity and stability. At present, detailed information about catalyst degradation can only be obtained using IL and *in situ* STEM. Catalyst–support interactions remain difficult to examine directly, however, the use of 4D STEM to reveal more about these interactions is a promising prospect. Thin window liquid cell design improvements for *in situ* TEM, reducing beam damage effects, and increasing the efficiency of acquisition and reconstruction in tomography are just some of the areas where boundaries can be pushed. Despite this, (S)TEM is regularly and effectively used in electrocatalysis studies and can be regarded as an essential catalyst characterization technique. It should once more be emphasized that interpretation of (S)TEM images and data should be done cautiously considering that results are collected locally, from a small area of a sample. For this reason, complementary information using bulk analytical methods is always desirable, such as crystallographic data from XRD or chemical composition analysis from ICP-OES or ICP-MS.

## Author contributions

Conceptualization, L. B., F. R. Z. and N. H.; writing – original draft, L. B., F. R. Z. and N. H.; writing – review & editing, L. B., F. R. Z. and N. H.; funding acquisition, N. H.; supervision, F. R. Z. and N. H.

## Conflicts of interest

There are no conflicts to declare.

## Supplementary Material

## References

[cit1] Debe M. K. (2012). Electrocatalyst Approaches and Challenges for Automotive Fuel Cells. Nature.

[cit2] Shao M., Chang Q., Dodelet J.-P., Chenitz R. (2016). Recent Advances in Electrocatalysts for Oxygen Reduction Reaction. Chem. Rev..

[cit3] Fuel Cells: Data, Facts and Figures, ed. P. D. Stolten, D. R. C. Samsun and D. N. Garland, Wiley-VCH Verlag GmbH & Co. KGaA, Weinheim, Germany, 2016

[cit4] Hubert M. A., King L. A., Jaramillo T. F. (2022). Evaluating the Case for Reduced Precious Metal Catalysts in Proton Exchange Membrane Electrolyzers. ACS Energy Lett..

[cit5] Yang J., Yang J., Ying J. Y. (2012). Morphology and Lateral Strain Control of Pt Nanoparticles via Core–Shell Construction Using Alloy AgPd Core Toward Oxygen Reduction Reaction. ACS Nano.

[cit6] Mott D., Luo J., Njoki P. N., Lin Y., Wang L., Zhong C.-J. (2007). Synergistic Activity of Gold-Platinum Alloy Nanoparticle Catalysts. Catal. Today.

[cit7] Jayasayee K., Veen J. A. R. V., Manivasagam T. G., Celebi S., Hensen E. J. M., de Bruijn F. A. (2012). Oxygen Reduction Reaction (ORR) Activity and Durability of Carbon Supported PtM (Co, Ni, Cu) Alloys: Influence of Particle Size and Non-Noble Metals. Appl. Catal., B.

[cit8] Zhang J., Vukmirovic M. B., Xu Y., Mavrikakis M., Adzic R. R. (2005). Controlling the Catalytic Activity of Platinum-Monolayer Electrocatalysts for Oxygen Reduction with Different Substrates. Angew. Chem., Int. Ed..

[cit9] Mazumder V., Chi M., More K. L., Sun S. (2010). Synthesis and Characterization of Multimetallic Pd/Au and Pd/Au/FePt Core/Shell Nanoparticles. Angew. Chem., Int. Ed..

[cit10] Matin M. A., Lee J., Kim G. W., Park H.-U., Cha B. J., Shastri S., Kim G., Kim Y.-D., Kwon Y.-U., Petkov V. (2020). Morphing Mncore@Ptshell Nanoparticles: Effects of Core Structure on the ORR Performance of Pt Shell. Appl. Catal., B.

[cit11] Eriksson B., Montserrat-Sisó G., Brown R., Skála T., Wreland Lindström R., Lindbergh G., Wickman B., Lagergren C. (2021). Enhanced Oxygen Reduction Activity with Rare Earth Metal Alloy Catalysts in Proton Exchange Membrane Fuel Cells. Electrochim. Acta.

[cit12] Zhao L., Guo Y., Fu C., Luo L., Wei G., Shen S., Zhang J. (2021). Electrodeposited PtNi Nanoparticles towards Oxygen Reduction Reaction: A Study on Nucleation and Growth Mechanism. Chin. J. Catal..

[cit13] Wang J. X., Ma C., Choi Y., Su D., Zhu Y., Liu P., Si R., Vukmirovic M. B., Zhang Y., Adzic R. R. (2011). Kirkendall Effect and Lattice Contraction in Nanocatalysts: A New Strategy to Enhance Sustainable Activity. J. Am. Chem. Soc..

[cit14] Lopez-Haro M., Dubau L., Guétaz L., Bayle-Guillemaud P., Chatenet M., André J., Caqué N., Rossinot E., Maillard F. (2014). Atomic-Scale Structure and Composition of Pt_3_Co/C Nanocrystallites during Real PEMFC Operation: A STEM–EELS Study. Appl. Catal., B.

[cit15] Su L., Jia W., Li C.-M., Lei Y. (2014). Mechanisms for Enhanced Performance of Platinum-Based Electrocatalysts in Proton Exchange Membrane Fuel Cells. ChemSusChem.

[cit16] Yu T., Kim D. Y., Zhang H., Xia Y. (2011). Platinum Concave Nanocubes with High–Index Facets and Their Enhanced Activity for Oxygen Reduction Reaction. Angew. Chem., Int. Ed..

[cit17] Zhang X., Li H., Yang J., Lei Y., Wang C., Wang J., Tang Y., Mao Z. (2021). Recent Advances in Pt-Based Electrocatalysts for PEMFCs. RSC Adv..

[cit18] Chen L., Zhang X., Chen A., Yao S., Hu X., Zhou Z. (2022). Targeted Design of Advanced Electrocatalysts by Machine Learning. Chin. J. Catal..

[cit19] Li Z., Wang S., Xin H. (2018). Toward Artificial Intelligence in Catalysis. Nat. Catal..

[cit20] Lin Y., Zhou M., Tai X., Li H., Han X., Yu J. (2021). Analytical Transmission Electron Microscopy for Emerging Advanced Materials. Matter.

[cit21] Moriau L. J., Hrnjić A., Pavlišič A., Kamšek A. R., Petek U., Ruiz-Zepeda F., Šala M., Pavko L., Šelih V. S., Bele M., Jovanovič P., Gatalo M., Hodnik N. (2021). Resolving the Nanoparticles’ Structure-Property Relationships at the Atomic Level: A Study of Pt-Based Electrocatalysts. iScience.

[cit22] Huang X., Zhao Z., Cao L., Chen Y., Zhu E., Lin Z., Li M., Yan A., Zettl A., Wang Y. M., Duan X., Mueller T., Huang Y. (2015). High-Performance Transition Metal–Doped Pt _3_ Ni Octahedra for Oxygen Reduction Reaction. Science.

[cit23] Zhang L., Roling L. T., Wang X., Vara M., Chi M., Liu J., Choi S.-I., Park J., Herron J. A., Xie Z., Mavrikakis M., Xia Y. (2015). Platinum-Based Nanocages with Subnanometer-Thick Walls and Well-Defined, Controllable Facets. Science.

[cit24] Yang Y., Xiong Y., Zeng R., Lu X., Krumov M., Huang X., Xu W., Wang H., DiSalvo F. J., Brock J. D., Muller D. A., Abruña H. D. (2021). Operando Methods in Electrocatalysis. ACS Catal..

[cit25] Williams D. B. and Carter C. B., Transmission Electron Microscopy, Springer US, Boston, MA, 2009

[cit26] Hosokawa F., Sawada H., Kondo Y., Takayanagi K., Suenaga K. (2013). Development of Cs and Cc Correctors for Transmission Electron Microscopy. Microscopy.

[cit27] Sawada H., Tomita T., Naruse M., Honda T., Hambridge P., Hartel P., Haider M., Hetherington C., Doole R., Kirkland A., Hutchison J., Titchmarsh J., Cockayne D. (2005). Experimental Evaluation of a Spherical Aberration-Corrected TEM and STEM. Microscopy.

[cit28] Li J., Xia W., Guo Y., Qi R., Xu X., Jiang D., Wang T., Sugahara Y., He J., Yamauchi Y. (2023). Surface Curvature Effect on Single-Atom Sites for the Oxygen Reduction Reaction: A Model of Mesoporous MOF-Derived Carbon. Chem. Eng. J..

[cit29] Li J., Xia W., Tang J., Gao Y., Jiang C., Jia Y., Chen T., Hou Z., Qi R., Jiang D., Asahi T., Xu X., Wang T., He J., Yamauchi Y. (2022). Metal–Organic Framework-Derived Graphene Mesh: A Robust Scaffold for Highly Exposed Fe–N _4_ Active Sites toward an Excellent Oxygen Reduction Catalyst in Acid Media. J. Am. Chem. Soc..

[cit30] Cheng X., Wang Y., Lu Y., Zheng L., Sun S., Li H., Chen G., Zhang J. (2022). Single-Atom Alloy with Pt-Co Dual Sites as an Efficient Electrocatalyst for Oxygen Reduction Reaction. Appl. Catal., B.

[cit31] Calle-Vallejo F., Tymoczko J., Colic V., Vu Q. H., Pohl M. D., Morgenstern K., Loffreda D., Sautet P., Schuhmann W., Bandarenka A. S. (2015). Finding Optimal Surface Sites on Heterogeneous Catalysts by Counting Nearest Neighbors. Science.

[cit32] Nesselberger M., Ashton S., Meier J. C., Katsounaros I., Mayrhofer K. J. J., Arenz M. (2011). The Particle Size Effect on the Oxygen Reduction Reaction Activity of Pt Catalysts: Influence of Electrolyte and Relation to Single Crystal Models. J. Am. Chem. Soc..

[cit33] Stephens I. E. L., Elias J. S., Shao-Horn Y. (2015). The Importance of Being Together. Science.

[cit34] Viswanathan V., Wang F. Y.-F. (2012). Theoretical Analysis of the Effect of Particle Size and Support on the Kinetics of Oxygen Reduction Reaction on Platinum Nanoparticles. Nanoscale.

[cit35] Perez-Alonso F. J., McCarthy D. N., Nierhoff A., Hernandez-Fernandez P., Strebel C., Stephens I. E. L., Nielsen J. H., Chorkendorff I. (2012). The Effect of Size on the Oxygen Electroreduction Activity of Mass-Selected Platinum Nanoparticles. Angew. Chem., Int. Ed..

[cit36] Yano H., Watanabe M., Iiyama A., Uchida H. (2016). Particle-Size Effect of Pt Cathode Catalysts on Durability in Fuel Cells. Nano Energy.

[cit37] Jovanovič P., Petek U., Hodnik N., Ruiz-Zepeda F., Gatalo M., Šala M., Šelih V. S., Fellinger T. P., Gaberšček M. (2017). Importance of Non-Intrinsic Platinum Dissolution in Pt/C Composite Fuel Cell Catalysts. Phys. Chem. Chem. Phys..

[cit38] Marković N. M., Schmidt T. J., Stamenković V., Ross P. N. (2001). Oxygen Reduction Reaction on Pt and Pt Bimetallic Surfaces: A Selective Review. Fuel Cells.

[cit39] Guo S., Zhang S., Sun S. (2013). Tuning Nanoparticle Catalysis for the Oxygen Reduction Reaction. Angew. Chem., Int. Ed..

[cit40] Mazumder V., Lee Y., Sun S. (2010). Recent Development of Active Nanoparticle Catalysts for Fuel Cell Reactions. Adv. Funct. Mater..

[cit41] Sánchez-Sánchez C. M., Solla-Gullón J., Vidal-Iglesias F. J., Aldaz A., Montiel V., Herrero E. (2010). Imaging Structure Sensitive Catalysis on Different Shape-Controlled Platinum Nanoparticles. J. Am. Chem. Soc..

[cit42] Wang C., Daimon H., Onodera T., Koda T., Sun S. (2008). A General Approach to the Size- and Shape-Controlled Synthesis of Platinum Nanoparticles and Their Catalytic Reduction of Oxygen. Angew. Chem..

[cit43] Divi S., Chatterjee A. (2018). Generalized Nano-Thermodynamic
Model for Capturing Size-Dependent Surface Segregation in Multi-Metal Alloy Nanoparticles. RSC Adv..

[cit44] Gan L., Cui C., Heggen M., Dionigi F., Rudi S., Strasser P. (2014). Element-Specific Anisotropic Growth of Shaped Platinum Alloy Nanocrystals. Science.

[cit45] Chen C., Kang Y., Huo Z., Zhu Z., Huang W., Xin H. L., Snyder J. D., Li D., Herron J. A., Mavrikakis M., Chi M., More K. L., Li Y., Markovic N. M., Somorjai G. A., Yang P., Stamenkovic V. R. (2014). Highly Crystalline Multimetallic Nanoframes with Three-Dimensional Electrocatalytic Surfaces. Science.

[cit46] Li M., Zhao Z., Cheng T., Fortunelli A., Chen C.-Y., Yu R., Zhang Q., Gu L., Merinov B. V., Lin Z., Zhu E., Yu T., Jia Q., Guo J., Zhang L., Goddard W. A., Huang Y., Duan X. (2016). Ultrafine Jagged Platinum Nanowires Enable Ultrahigh Mass Activity for the Oxygen Reduction Reaction. Science.

[cit47] Bu L., Zhang N., Guo S., Zhang X., Li J., Yao J., Wu T., Lu G., Ma J.-Y., Su D., Huang X. (2016). Biaxially Strained PtPb/Pt Core/Shell Nanoplate Boosts Oxygen Reduction Catalysis. Science.

[cit48] Stephens I. E. L., Rossmeisl J., Chorkendorff I. (2016). Toward Sustainable Fuel Cells. Science.

[cit49] Zhang P., Lu Y.-R., Hsu C.-S., Xue H.-G., Chan T.-S., Suen N.-T., Chen H. M. (2020). Electronic Structure Inspired a Highly Robust Electrocatalyst for the Oxygen-Evolution Reaction. Chem. Commun..

[cit50] Li X., Chen T., Yang B., Xiang Z. (2023). Fundamental Understanding of Electronic Structure in FeN _4_ Site on Electrocatalytic Activity via Dz^2^ –Orbital–Driven Charge Tuning for Acidic Oxygen Reduction. Angew. Chem., Int. Ed..

[cit51] Yin C., Li Q., Zheng J., Ni Y., Wu H., Kjøniksen A.-L., Liu C., Lei Y., Zhang Y. (2022). Progress in Regulating Electronic Structure Strategies on Cu-Based Bimetallic Catalysts for CO_2_ Reduction Reaction. Adv. Powder Mater..

[cit52] Zhang S., Zhang X., Jiang G., Zhu H., Guo S., Su D., Lu G., Sun S. (2014). Tuning Nanoparticle Structure and Surface Strain for Catalysis Optimization. J. Am. Chem. Soc..

[cit53] Luo M., Guo S. (2017). Strain-Controlled Electrocatalysis on Multimetallic Nanomaterials. Nat. Rev. Mater..

[cit54] Mistry H., Varela A. S., Kühl S., Strasser P., Cuenya B. R. (2016). Nanostructured Electrocatalysts with Tunable Activity and Selectivity. Nat. Rev. Mater..

[cit55] Strasser P., Koh S., Anniyev T., Greeley J., More K., Yu C., Liu Z., Kaya S., Nordlund D., Ogasawara H., Toney M. F., Nilsson A. (2010). Lattice-Strain Control of the Activity in Dealloyed Core–Shell Fuel Cell Catalysts. Nat. Chem..

[cit56] Jo H., Wi D. H., Lee T., Kwon Y., Jeong C., Lee J., Baik H., Pattison A. J., Theis W., Ophus C., Ercius P., Lee Y.-L., Ryu S., Han S. W., Yang Y. (2022). Direct Strain Correlations at the Single-Atom Level in Three-Dimensional Core-Shell Interface Structures. Nat. Commun..

[cit57] Gan L., Yu R., Luo J., Cheng Z., Zhu J. (2012). Lattice Strain Distributions in Individual Dealloyed Pt–Fe Catalyst Nanoparticles. J. Phys. Chem. Lett..

[cit58] Galindo P. L., Kret S., Sanchez A. M., Laval J.-Y., Yáñez A., Pizarro J., Guerrero E., Ben T., Molina S. I. (2007). The Peak Pairs Algorithm for Strain Mapping from HRTEM Images. Ultramicroscopy.

[cit59] Béché A., Rouvière J. L., Barnes J. P., Cooper D. (2011). Dark Field Electron Holography for Strain Measurement. Ultramicroscopy.

[cit60] Ruiz-Zepeda F., Gatalo M., Pavlišič A., Dražić G., Jovanovič P., Bele M., Gaberšček M., Hodnik N. (2019). Atomically Resolved Anisotropic Electrochemical Shaping of Nano-Electrocatalyst. Nano Lett..

[cit61] Hÿtch M. J., Snoeck E., Kilaas R. (1998). Quantitative Measurement of Displacement and Strain Fields from HREM Micrographs. Ultramicroscopy.

[cit62] Gamler J. T. L., Leonardi A., Sang X., Koczkur K. M., Unocic R. R., Engel M., Skrabalak S. E. (2020). Effect of Lattice Mismatch and Shell Thickness on Strain in Core@shell Nanocrystals. Nanoscale Adv..

[cit63] Béché A., Rouvière J. L., Barnes J. P., Cooper D. (2013). Strain Measurement at the Nanoscale: Comparison between Convergent Beam Electron Diffraction, Nano-Beam Electron Diffraction, High Resolution Imaging and Dark Field Electron Holography. Ultramicroscopy.

[cit64] Padgett E., Holtz M. E., Cueva P., Shao Y.-T., Langenberg E., Schlom D. G., Muller D. A. (2020). The Exit-Wave Power-Cepstrum Transform for Scanning Nanobeam Electron Diffraction: Robust Strain Mapping at Subnanometer Resolution and Subpicometer Precision. Ultramicroscopy.

[cit65] Houdellier F., Roucau C., Clément L., Rouvière J. L., Casanove M. J. (2006). Quantitative Analysis of HOLZ Line Splitting in CBED Patterns of Epitaxially Strained Layers. Ultramicroscopy.

[cit66] Hÿtch M. J., Minor A. M. (2014). Observing and Measuring Strain in Nanostructures and Devices with Transmission Electron Microscopy. MRS Bull..

[cit67] Science of Microscopy, ed. P. W. Hawkes and J. C. H. Spence, Springer New York, New York, NY, 2007

[cit68] Krivanek O. L., Dellby N., Hachtel J. A., Idrobo J.-C., Hotz M. T., Plotkin-Swing B., Bacon N. J., Bleloch A. L., Corbin G. J., Hoffman M. V., Meyer C. E., Lovejoy T. C. (2019). Progress in Ultrahigh Energy Resolution EELS. Ultramicroscopy.

[cit69] Egerton R. F. , Electron Energy-Loss Spectroscopy in the Electron Microscope, Springer US, Boston, MA, 2011

[cit70] Yoo T. Y., Yoo J. M., Sinha A. K., Bootharaju M. S., Jung E., Lee H. S., Lee B.-H., Kim J., Antink W. H., Kim Y. M., Lee J., Lee E., Lee D. W., Cho S.-P., Yoo S. J., Sung Y.-E., Hyeon T. (2020). Direct Synthesis of Intermetallic Platinum–Alloy Nanoparticles Highly Loaded on Carbon Supports for Efficient Electrocatalysis. J. Am. Chem. Soc..

[cit71] Lv H., Zheng Y., Wang Y., Wang J., Liu B., Qiao Z. (2023). Ordered Mesoporous Intermetallic Ga–Pt Nanoparticles: Phase–Controlled Synthesis and Performance in Oxygen Reduction Electrocatalysis. Angew. Chem., Int. Ed..

[cit72] Peng Z., Wu J., Yang H. (2010). Synthesis and Oxygen Reduction Electrocatalytic Property of Platinum Hollow and Platinum-on-Silver Nanoparticles. Chem. Mater..

[cit73] Sasaki K., Naohara H., Choi Y., Cai Y., Chen W.-F., Liu P., Adzic R. R. (2012). Highly Stable Pt Monolayer on PdAu Nanoparticle Electrocatalysts for the Oxygen Reduction Reaction. Nat. Commun..

[cit74] Hunt S. T., Milina M., Alba-Rubio A. C., Hendon C. H., Dumesic J. A., Roman-Leshkov Y. (2016). Self-Assembly of Noble Metal Monolayers on Transition Metal Carbide Nanoparticle Catalysts. Science.

[cit75] Xie S., Choi S.-I., Lu N., Roling L. T., Herron J. A., Zhang L., Park J., Wang J., Kim M. J., Xie Z., Mavrikakis M., Xia Y. (2014). Atomic Layer-by-Layer Deposition of Pt on Pd Nanocubes for Catalysts with Enhanced Activity and Durability toward Oxygen Reduction. Nano Lett..

[cit76] Hamon A.-L., Verbeeck J., Schryvers D., Benedikt J., Sanden R. M. C. M. v. d. (2004). ELNES Study of Carbon K-Edge Spectra of Plasma Deposited Carbon Films. J. Mater. Chem..

[cit77] Schmid H. K., Mader W. (2006). Oxidation States of Mn and Fe in Various Compound Oxide Systems. Micron.

[cit78] Daulton T. L., Little B. J. (2006). Determination of Chromium Valence over the Range Cr(0)–Cr(vi) by Electron Energy Loss Spectroscopy. Ultramicroscopy.

[cit79] Cui C., Gan L., Heggen M., Rudi S., Strasser P. (2013). Compositional Segregation in Shaped Pt Alloy Nanoparticles and Their Structural Behaviour during Electrocatalysis. Nat. Mater..

[cit80] Yu K., Hart J. L., Xie J., Taheri M. L., Ferreira P. (2023). A Novel Approach to Identify the Ionomer Phase in PEMFC by EELS. Nano Energy.

[cit81] Scheiba F., Benker N., Kunz U., Roth C., Fuess H. (2008). Electron Microscopy Techniques for the Analysis of the Polymer Electrolyte Distribution in Proton Exchange Membrane Fuel Cells. J. Power Sources.

[cit82] Hodnik N., Cherevko S. (2019). Spot the Difference at the Nanoscale: Identical Location Electron Microscopy in Electrocatalysis. Curr. Opin. Electrochem..

[cit83] Hodnik N., Dehm G., Mayrhofer K. J. J. (2016). Importance and Challenges of Electrochemical in Situ Liquid Cell Electron Microscopy for Energy Conversion Research. Acc. Chem. Res..

[cit84] Borup R., Meyers J., Pivovar B., Kim Y. S., Mukundan R., Garland N., Myers D., Wilson M., Garzon F., Wood D., Zelenay P., More K., Stroh K., Zawodzinski T., Boncella J., McGrath J. E., Inaba M., Miyatake K., Hori M., Ota K., Ogumi Z., Miyata S., Nishikata A., Siroma Z., Uchimoto Y., Yasuda K., Kimijima K., Iwashita N. (2007). Scientific Aspects of Polymer Electrolyte Fuel Cell Durability and Degradation. Chem. Rev..

[cit85] Mayrhofer K. J. J., Meier J. C., Ashton S. J., Wiberg G. K. H., Kraus F., Hanzlik M., Arenz M. (2008). Fuel Cell Catalyst Degradation on the Nanoscale. Electrochem. Commun..

[cit86] Mayrhofer K. J. J., Ashton S. J., Meier J. C., Wiberg G. K. H., Hanzlik M., Arenz M. (2008). Non-Destructive Transmission Electron Microscopy Study of Catalyst Degradation under Electrochemical Treatment. J. Power Sources.

[cit87] Shao-Horn Y., Sheng W. C., Chen S., Ferreira P. J., Holby E. F., Morgan D. (2007). Instability of Supported Platinum Nanoparticles in Low-Temperature Fuel Cells. Top. Catal..

[cit88] Sheng W., Chen S., Vescovo E., Shao-Horn Y. (2011). Size Influence on the Oxygen Reduction Reaction Activity and Instability of Supported Pt Nanoparticles. J. Electrochem. Soc..

[cit89] Ferreira P. J., la O′ G. J., Shao-Horn Y., Morgan D., Makharia R., Kocha S., Gasteiger H. A. (2005). Instability of Pt/C Electrocatalysts in Proton Exchange Membrane Fuel Cells. J. Electrochem. Soc..

[cit90] Meier J. C., Galeano C., Katsounaros I., Topalov A. A., Kostka A., Schüth F., Mayrhofer K. J. J. (2012). Degradation Mechanisms of Pt/C Fuel Cell Catalysts under Simulated Start–Stop Conditions. ACS Catal..

[cit91] Schlögl K., Mayrhofer K. J. J., Hanzlik M., Arenz M. (2011). Identical-Location TEM Investigations of Pt/C Electrocatalyst Degradation at Elevated Temperatures. J. Electroanal. Chem..

[cit92] Schulenburg H., Schwanitz B., Linse N., Scherer G. G., Wokaun A., Krbanjevic J., Grothausmann R., Manke I. (2011). 3D Imaging of Catalyst Support Corrosion in Polymer Electrolyte Fuel Cells. J. Phys. Chem. C.

[cit93] Meier J. C., Katsounaros I., Galeano C., Bongard H. J., Topalov A. A., Kostka A., Karschin A., Schüth F., Mayrhofer K. J. J. (2012). Stability Investigations of Electrocatalysts on the Nanoscale. Energy Environ. Sci..

[cit94] Rasouli S., Myers D., Kariuki N., Higashida K., Nakashima N., Ferreira P. (2019). Electrochemical Degradation of Pt–Ni Nanocatalysts: An Identical Location Aberration-Corrected Scanning Transmission Electron Microscopy Study. Nano Lett..

[cit95] Perez-Alonso F. J., Elkjær C. F., Shim S. S., Abrams B. L., Stephens I. E. L., Chorkendorff I. (2011). Identical Locations Transmission Electron Microscopy Study of Pt/C Electrocatalyst Degradation during Oxygen Reduction Reaction. J. Power Sources.

[cit96] Jovanovič P., Ruiz-Zepeda F., Šala M., Hodnik N. (2018). Atomic Scale Insights into Electrochemical Dissolution of Janus Pt–SnO _2_ Nanoparticles in the Presence of Ethanol in Acidic Media: An IL-STEM and EFC–ICP–MS Study. J. Phys. Chem. C.

[cit97] Hrnjić A., Ruiz-Zepeda F., Gaberšček M., Bele M., Suhadolnik L., Hodnik N., Jovanovič P. (2020). Modified Floating Electrode Apparatus for Advanced Characterization of Oxygen Reduction Reaction Electrocatalysts. J. Electrochem. Soc..

[cit98] Hrnjic A., Kamšek A. R., Pavlišič A., Šala M., Bele M., Moriau L., Gatalo M., Ruiz-Zepeda F., Jovanovič P., Hodnik N. (2021). Observing, Tracking and Analysing Electrochemically Induced Atomic-Scale Structural Changes of an Individual Pt-Co Nanoparticle as a Fuel Cell Electrocatalyst by Combining Modified Floating Electrode and Identical Location Electron Microscopy. Electrochim. Acta.

[cit99] Bele M., Podboršek G. K., Lončar A., Jovanovič P., Hrnjić A., Marinko Ž, Kovač J., Surca A. K., Kamšek A. R., Dražić G., Hodnik N., Suhadolnik L. (2023). “Nano Lab" Advanced Characterization Platform for Studying Electrocatalytic Iridium Nanoparticles Dispersed on TiO_*x*_N_*y*_ Supports Prepared on Ti Transmission Electron Microscopy Grids. ACS Appl. Nano Mater..

[cit100] Kamšek A. R., Ruiz-Zepeda F., Pavlišič A., Hrnjić A., Hodnik N. (2022). Bringing into Play Automated Electron Microscopy Data Processing for Understanding Nanoparticulate Electrocatalysts’ Structure–Property Relationships. Curr. Opin. Electrochem..

[cit101] Mitchell D. R. G., Casillas G. (2016). Secondary Electron Imaging in an Aberration-Corrected STEM. Microsc. Today.

[cit102] Habas S. E., Lee H., Radmilovic V., Somorjai G. A., Yang P. (2007). Shaping Binary Metal Nanocrystals through Epitaxial Seeded Growth. Nat. Mater..

[cit103] Langille M. R., Zhang J., Personick M. L., Li S., Mirkin C. A. (2012). Stepwise Evolution of Spherical Seeds into 20-Fold Twinned Icosahedra. Science.

[cit104] Saghi Z., Midgley P. A. (2012). Electron Tomography in the (S)TEM: From Nanoscale Morphological Analysis to 3D Atomic Imaging. Annu. Rev. Mater. Res..

[cit105] Hodnik N., Jeyabharathi C., Meier J. C., Kostka A., Phani K. L., Rečnik A., Bele M., Hočevar S., Gaberšček M., Mayrhofer K. J. J. (2014). Effect of Ordering of PtCu _3_ Nanoparticle Structure on the Activity and Stability for the Oxygen Reduction Reaction. Phys. Chem. Chem. Phys..

[cit106] Arán-Ais R. M., Yu Y., Hovden R., Solla-Gullón J., Herrero E., Feliu J. M., Abruña H. D. (2015). Identical Location Transmission Electron Microscopy Imaging of Site-Selective Pt Nanocatalysts: Electrochemical Activation and Surface Disordering. J. Am. Chem. Soc..

[cit107] Padgett E., Andrejevic N., Liu Z., Kongkanand A., Gu W., Moriyama K., Jiang Y., Kumaraguru S., Moylan T. E., Kukreja R., Muller D. A. (2018). Editors’ Choice—Connecting Fuel Cell Catalyst Nanostructure and Accessibility Using Quantitative Cryo-STEM Tomography. J. Electrochem. Soc..

[cit108] Bals S., Goris B., De Backer A., Van Aert S., Van Tendeloo G. (2016). Atomic Resolution Electron Tomography. MRS Bull..

[cit109] Li Z., Xie Z., Zhang Y., Mu X., Xie J., Yin H.-J., Zhang Y.-W., Ophus C., Zhou J. (2023). Probing the Atomically Diffuse Interfaces in Pd@Pt Core-Shell Nanoparticles in Three Dimensions. Nat. Commun..

[cit110] Jo H., Wi D. H., Lee T., Kwon Y., Jeong C., Lee J., Baik H., Pattison A. J., Theis W., Ophus C., Ercius P., Lee Y.-L., Ryu S., Han S. W., Yang Y. (2022). Direct Strain Correlations at the Single-Atom Level in Three-Dimensional Core-Shell Interface Structures. Nat. Commun..

[cit111] De Backer A., Bals S., Van Aert S. (2023). A Decade of Atom-Counting in STEM: From the First Results toward Reliable 3D Atomic Models from a Single Projection. Ultramicroscopy.

[cit112] De Backer A., Zhang Z., van den Bos K. H. W., Bladt E., Sánchez-Iglesias A., Liz-Marzán L. M., Nellist P. D., Bals S., Van Aert S. (2022). Element Specific Atom Counting at the Atomic Scale by Combining High Angle Annular Dark Field Scanning Transmission Electron Microscopy and Energy Dispersive X–ray Spectroscopy. Small Methods.

[cit113] Skorikov A., Batenburg K. J., Bals S. (2023). Analysis of 3D Elemental Distribution in Nanomaterials: Towards Higher Throughput and Dose Efficiency. J. Microsc..

[cit114] Albrecht W., Bals S. (2020). Fast Electron Tomography for Nanomaterials. J. Phys. Chem. C.

[cit115] da Silva A., David T., Saghi Z., Guetaz L. (2021). Multiple ADF-STEM Towards the Optimization of Electron Tomography Reconstructions of Pt/C Fuel Cell Catalyst Nanostructures. Microsc. Microanal..

[cit116] Amichi L., Yu H., Zachman M. J., Ziabari A., Arregui-Mena D., Guetaz L., David T., Saghi Z., Ghorbel A., Cullen D. A. (2023). Investigation of Nanoparticle Degradation in Hydrogen Fuel Cell Systems through Automated Electron Microscopy. Microsc. Microanal..

[cit117] Girod R., Lazaridis T., Gasteiger H. A., Tileli V. (2023). Three-Dimensional Nanoimaging of Fuel Cell Catalyst Layers. Nat. Catal..

[cit118] Ophus C. (2019). Four-Dimensional Scanning Transmission Electron Microscopy (4D-STEM): From Scanning Nanodiffraction to Ptychography and Beyond. Microsc. Microanal..

[cit119] Wang S., Eldred T. B., Smith J. G., Gao W. (2022). AutoDisk: Automated Diffraction Processing and Strain Mapping in 4D-STEM. Ultramicroscopy.

[cit120] Mukherjee D., Gamler J. T. L., Skrabalak S. E., Unocic R. R. (2020). Lattice Strain Measurement of Core@Shell Electrocatalysts with 4D Scanning Transmission Electron Microscopy Nanobeam Electron Diffraction. ACS Catal..

[cit121] Ophus C., Zeltmann S. E., Bruefach A., Rakowski A., Savitzky B. H., Minor A. M., Scott M. C. (2022). Automated Crystal Orientation Mapping in Py4DSTEM Using Sparse Correlation Matching. Microsc. Microanal..

[cit122] Rauch E. F., Portillo J., Nicolopoulos S., Bultreys D., Rouvimov S., Moeck P. (2010). Automated Nanocrystal Orientation and Phase Mapping in the Transmission Electron Microscope on the Basis of Precession Electron Diffraction. Z. Kristallogr..

[cit123] Martis J., Susarla S., Rayabharam A., Su C., Paule T., Pelz P., Huff C., Xu X., Li H.-K., Jaikissoon M., Chen V., Pop E., Saraswat K., Zettl A., Aluru N. R., Ramesh R., Ercius P., Majumdar A. (2023). Imaging the Electron Charge Density in Monolayer MoS_2_ at the Ångstrom Scale. Nat. Commun..

[cit124] Solymosi F. (1968). Importance of the Electric Properties of Supports in the Carrier Effect. Catal. Rev..

[cit125] Pan C.-J., Tsai M.-C., Su W.-N., Rick J., Akalework N. G., Agegnehu A. K., Cheng S.-Y., Hwang B.-J. (2017). Tuning/Exploiting Strong Metal-Support Interaction (SMSI) in Heterogeneous Catalysis. J. Taiwan Inst. Chem. Eng..

[cit126] Mejía H., van Deelen C., de Jong T. W., P K. (2018). Activity Enhancement of Cobalt Catalysts by Tuning Metal-Support Interactions. Nat. Commun..

[cit127] Zachman M. J., Fung V., Polo-Garzon F., Cao S., Moon J., Huang Z., Jiang D., Wu Z., Chi M. (2022). Measuring and Directing Charge Transfer in Heterogenous Catalysts. Nat. Commun..

[cit128] Jiang Y., Chen Z., Han Y., Deb P., Gao H., Xie S., Purohit P., Tate M. W., Park J., Gruner S. M., Elser V., Muller D. A. (2018). Electron Ptychography of 2D Materials to Deep Sub-Ångström Resolution. Nature.

[cit129] Li G., Zhang H., Han Y. (2022). 4D-STEM Ptychography for Electron-Beam-Sensitive Materials. ACS Cent. Sci..

[cit130] Yang H., Rutte R. N., Jones L., Simson M., Sagawa R., Ryll H., Huth M., Pennycook T. J., Green M. L. H., Soltau H., Kondo Y., Davis B. G., Nellist P. D. (2016). Simultaneous Atomic-Resolution Electron Ptychography and Z-Contrast Imaging of Light and Heavy Elements in Complex Nanostructures. Nat. Commun..

[cit131] Gao S., Wang P., Zhang F., Martinez G. T., Nellist P. D., Pan X., Kirkland A. I. (2017). Electron Ptychographic Microscopy for Three-Dimensional Imaging. Nat. Commun..

[cit132] Wang B., McComb D. W. (2023). Phase Imaging in Scanning Transmission Electron Microscopy Using Bright-Field Balanced Divergency Method. Ultramicroscopy.

[cit133] Ross F. M. (2015). Opportunities and Challenges in Liquid Cell Electron Microscopy. Science.

[cit134] Yang Y., Feijóo J., Briega-Martos V., Li Q., Krumov M., Merkens S., De Salvo G., Chuvilin A., Jin J., Huang H., Pollock C. J., Salmeron M. B., Wang C., Muller D. A., Abruña H. D., Yang P. (2023). Operando Methods: A New Era of Electrochemistry. Curr. Opin. Electrochem..

[cit135] Ruska E. (1942). Beitrag Zur übermikroskopischen Abbildung Bei Höheren Drucken. Kolloid-Z..

[cit136] Abrams I. M., McBain J. W. (1944). A Closed Cell for Electron Microscopy. Science.

[cit137] de Jonge N., Houben L., Dunin-Borkowski R. E., Ross F. M. (2018). Resolution and Aberration Correction in Liquid Cell Transmission Electron Microscopy. Nat. Rev. Mater..

[cit138] Williamson M. J., Tromp R. M., Vereecken P. M., Hull R., Ross F. M. (2003). Dynamic Microscopy of Nanoscale Cluster Growth at the Solid–Liquid Interface. Nat. Mater..

[cit139] Beermann V., Holtz M. E., Padgett E., de Araujo J. F., Muller D. A., Strasser P. (2019). Real-Time Imaging of Activation and Degradation of Carbon Supported Octahedral Pt–Ni Alloy Fuel Cell Catalysts at the Nanoscale Using in Situ Electrochemical Liquid Cell STEM. Energy Environ. Sci..

[cit140] Gong M., Xiao D., Deng Z., Zhang R., Xia W., Zhao T., Liu X., Shen T., Hu Y., Lu Y., Zhao X., Xin H., Wang D. (2021). Structure Evolution of PtCu Nanoframes from Disordered to Ordered for the Oxygen Reduction Reaction. Appl. Catal., B.

[cit141] Liao H.-G., Zherebetskyy D., Xin H., Czarnik C., Ercius P., Elmlund H., Pan M., Wang L.-W., Zheng H. (2014). Facet Development during Platinum Nanocube Growth. Science.

[cit142] Wulff G. (1901). XXV. Zur Frage Der Geschwindigkeit Des Wachsthums Und Der Auflösung Der Krystallflächen. Z. Kristallogr. - Cryst. Mater..

[cit143] Yang Y., Shao Y.-T., Lu X., Yang Y., Ko H.-Y., DiStasio R. A., DiSalvo F. J., Muller D. A., Abruña H. D. (2022). Elucidating Cathodic Corrosion Mechanisms with Operando Electrochemical Transmission Electron Microscopy. J. Am. Chem. Soc..

[cit144] Liao H.-G., Cui L., Whitelam S., Zheng H. (2012). Real-Time Imaging of Pt _3_ Fe Nanorod Growth in Solution. Science.

[cit145] Clark N., Kelly D. J., Zhou M., Zou Y.-C., Myung C. W., Hopkinson D. G., Schran C., Michaelides A., Gorbachev R., Haigh S. J. (2022). Tracking Single Adatoms in Liquid in a Transmission Electron Microscope. Nature.

[cit146] Yang J., Alam S. B., Yu L., Chan E., Zheng H. (2019). Dynamic Behavior of Nanoscale Liquids in Graphene Liquid Cells Revealed by in Situ Transmission Electron Microscopy. Micron.

[cit147] M W. A. R. D., Theobald R., Sharman B., Boyes J., Gai E. D., L P. (2018). Direct Observations of Dynamic PtCo Interactions in Fuel Cell Catalyst Precursors at the Atomic Level Using E(S)TEM. J. Microsc..

[cit148] Zhang C., Firestein K. L., Fernando J. F. S., Siriwardena D., Treifeldt J. E., Golberg D. (2020). Recent Progress of In Situ Transmission Electron Microscopy for Energy Materials. Adv. Mater..

[cit149] Unocic R. R., Jungjohann K. L., Mehdi B. L., Browning N. D., Wang C. (2020). In Situ Electrochemical Scanning/Transmission Electron Microscopy of Electrode–Electrolyte Interfaces. MRS Bull..

[cit150] Su D. (2017). Advanced Electron Microscopy Characterization of Nanomaterials for Catalysis. Green Energy Environ..

[cit151] Stricker E. A., Ke X., Wainright J. S., Unocic R. R., Savinell R. F. (2019). Current Density Distribution in Electrochemical Cells with Small Cell Heights and Coplanar Thin Electrodes as Used in Ec-S/TEM Cell Geometries. J. Electrochem. Soc..

[cit152] Ambrožič B., Prašnikar A., Hodnik N., Kostevšek N., Likozar B., Rožman K. Ž., Šturm S. (2019). Controlling the Radical-Induced Redox Chemistry inside a Liquid-Cell TEM. Chem. Sci..

[cit153] Han C., Islam M. T., Ni C. (2021). In Situ TEM of Electrochemical Incidents: Effects of Biasing and Electron Beam on Electrochemistry. ACS Omega.

[cit154] Yang Y., Shao Y.-T., Lu X., Yang Y., Ko H.-Y., DiStasio R. A., DiSalvo F. J., Muller D. A., Abruña H. D. (2022). Elucidating Cathodic Corrosion Mechanisms with Operando Electrochemical Transmission Electron Microscopy. J. Am. Chem. Soc..

[cit155] Reidy K., Thomsen J. D., Ross F. M. (2023). Perspectives on Ultra-High Vacuum Transmission Electron Microscopy of Dynamic Crystal Growth Phenomena. Prog. Mater. Sci..

[cit156] Qu J., Sui M., Li R. (2023). Recent Advances in *In situ* Transmission Electron Microscopy Techniques for Heterogeneous Catalysis. iScience.

[cit157] Kosari A., Zandbergen H., Tichelaar F., Visser P., Terryn H., Mol A. (2020). Application of In Situ Liquid Cell Transmission Electron Microscopy in Corrosion Studies: A Critical Review of Challenges and Achievements. Corrosion.

[cit158] Xie J., Li J., Mai W., Hong G. (2021). A Decade of Advanced Rechargeable Batteries Development Guided by in Situ Transmission Electron Microscopy. Nano Energy.

[cit159] Yang Y., Louisia S., Yu S., Jin J., Roh I., Chen C., Fonseca Guzman M. V., Feijóo J., Chen P.-C., Wang H., Pollock C. J., Huang X., Shao Y.-T., Wang C., Muller D. A., Abruña H. D., Yang P. (2023). Operando Studies Reveal Active Cu Nanograins for CO_2_ Electroreduction. Nature.

[cit160] Yang Y., Shao Y.-T., Jin J., Feijóo J., Roh I., Louisia S., Yu S., Fonseca Guzman M. V., Chen C., Muller D. A., Abruña H. D., Yang P. (2023). Operando Electrochemical Liquid-Cell Scanning Transmission Electron Microscopy (EC-STEM) Studies of Evolving Cu Nanocatalysts for CO _2_ Electroreduction. ACS Sustainable Chem. Eng..

[cit161] Grosse P., Yoon A., Rettenmaier C., Herzog A., Chee S. W., Cuenya R., Author B. (2021). Correction: Dynamic Transformations of Cubic Copper Catalysts during CO_2_ Electroreduction and Its Impact on Catalytic Selectivity. Nat. Commun..

[cit162] Kalinin S. V., Ophus C., Voyles P. M., Erni R., Kepaptsoglou D., Grillo V., Lupini A. R., Oxley M. P., Schwenker E., Chan M. K. Y., Etheridge J., Li X., Han G. G. D., Ziatdinov M., Shibata N., Pennycook S. J. (2022). Machine Learning in Scanning Transmission Electron Microscopy. Nat. Rev. Methods Primers.

[cit163] Leitherer A., Yeo B. C., Liebscher C. H., Ghiringhelli L. M. (2023). Automatic Identification of Crystal Structures and Interfaces via Artificial-Intelligence-Based Electron Microscopy. npj Comput. Mater..

[cit164] Crozier P. A., Morales A. M., Leibovich M., Mohan S., Haluai P., Tan M., Vincent J., Gilankar A., Wang Y., Fernandez-Granda C. (2023). The Impact of Artificial Intelligence on In Situ Electron Microscopy. Microsc. Microanal..

[cit165] Roccapriore K. M., Boebinger M. G., Klein J., Weile M., Ross F., Ziatdinov M., Unocic R. R., Kalinin S. V. (2023). AI-Enabled, Automation of Atomic Manipulation and Characterization in the STEM. Microsc. Microanal..

[cit166] Xin H. L., Wang C., Ji Z., Hu M., Kong L. (2023). A Universal Data Synthesizer to Enable AI4TEM. Microsc. Microanal..

